# Nicotine Overrides DNA Damage-Induced G_1_/S Restriction in Lung Cells

**DOI:** 10.1371/journal.pone.0018619

**Published:** 2011-04-29

**Authors:** Takashi Nishioka, Daisuke Yamamoto, Tongbo Zhu, Jinjin Guo, Sung-Hoon Kim, Chang Yan Chen

**Affiliations:** 1 Department of Radiation Oncology, Beth Israel Deaconess Medical Center, Harvard Medical School, Boston, Massachusetts, United States of America; 2 College of Oriental Medicine, Kyung Hee University, Seoul, Republic of Korea; Texas A&M University, United States of America

## Abstract

As an addictive substance, nicotine has been suggested to facilitate pro-survival activities (such as anchorage-independent growth or angiogenesis) and the establishment of drug resistance to anticancer therapy. Tobacco smoking consists of a variety of carcinogens [such as benzopyrene (BP) and nitrosamine derivatives] that are able to cause DNA double strand breaks. However, the effect of nicotine on DNA damage-induced checkpoint response induced by genotoxins remains unknown. In this study, we investigated the events occurred during G_1_ arrest induced by γ-radiation or BP in nicotine-treated murine or human lung epithelial cells. DNA synthesis was rapidly inhibited after exposure to γ-radiation or BP treatment, accompanied with the activation of DNA damage checkpoint. When these cells were co-treated with nicotine, the growth restriction was compromised, manifested by upregulation of cyclin D and A, and attenuation of Chk2 phosphorylation. Knockdown of cyclin D or Chk2 by the *siRNAs* blocked nicotine-mediated effect on DNA damage checkpoint activation. However, nicotine treatment appeared to play no role in nocodazole-induced mitotic checkpoint activation. Overall, our study presented a novel observation, in which nicotine is able to override DNA damage checkpoint activated by tobacco-related carcinogen BP or γ-irradiation. The results not only indicates the potentially important role of nicotine in facilitating the establishment of genetic instability to promote lung tumorigenesis, but also warrants a dismal prognosis for cancer patients who are smokers, heavily exposed second-hand smokers or nicotine users.

## Introduction

Tobacco smoke imposes a high risk for human malignancies, especially for lung cancer, because it contains more than 4000 components, most of which are well defined carcinogens [1], [2]. For example, benzopyrene or NNK/NNN [4-(methylnitrosamino)-1-(3-pyridyl)-1-butanone/N′-nitrosonornicotine) are the carcinogens in tobacco smoke and known to cause the generation of DNA adducts or mutating growth-related genes (like *p53* or *ras*) [3]–[6]. Although Nicotine is a major component in tobacco smoke and exists in high concentrations in the smokers, its function has been well documented as an addictive substance affecting the central nerve system [7], [8]. Recently, studies demonstrated that nicotinic acetylcholine receptor (nAChR) expresses in various types of non-neuronal cells, such as lung, mammary epithelial or vascular endothelial cells [9]–[11]. Nicotine, via interacting with the receptor, was shown to promote cell growth and angiogenesis in *in vitro* or *in vivo* experimental models [12]–[14].

DNA damage threats to cells because it may cause mutations, alterations of chromosomal structures and loss of genetic information. Cells possess a machinery to maintain the genomic integrity in response to genostresses [15], [16]. Under genotoxic conditions, cells do not progress into S or M phase before lesions are properly repaired by activating DNA damage checkpoint [15], [16]. The reduction of the sensitivity to genotoxic agents closely links to loss of the checkpoint function that permits cells a high rate of genomic adaptation to acquire a growth advantage. DNA damage checkpoint acts as a process of signal transduction to transmit information from damaged lesions to cell cycle regulators. In this process, ATM for double strand DNA breaks and ATR for single strand damages appear to be activated and further mediates a cascade of protein phosphorylation of effector checkpoint kinases (including Chk1 and Chk2) [17]–[20]. Under the influence of these checkpoint regulators, cells arrest at the G_1_/S or G_2_/M phases of the cell cycle, respectively [20], [21]. Although many components in tobacco smoke have been proved to be genotoxic, the combination effect of nicotine and tobacco-related genotoxic reagents or therapeutic radiation on DNA damage response remains unexplored.

Inhibition of the activity of cyclin D complexes is the key event to govern the G_1_/S checkpoint in cells [22], [23]. In this process, a rapid but transient response is elicited following cdc25A degradation, whereas a p21 or p53-mediated signaling supports a sustained reaction [22], [23]. After DNA double strand breaks, the ATM-mediated DNA damage checkpoint pathway is activated by autophosphorylation and activation of ATM, which in turn, phosphorylates Chk2 that often initiates the phosphorylation of several effector proteins, including phosphatases of the cdc25 family or BRCA1 [24], [25]. Dephosphorylation of cdc25A promotes the transition of cells from G_1_/S by removing the inhibitory phosphates on the cdk2/cyclinE complex [24], [25]. In the activation of DNA damage checkpoint, the phosphorylation of cdc25A by Chk2 causes its degradation, which blocks the function of G_1_/S regulators, leading to growth arrest [24], [25].

The irradiation with γ ray has been implemented clinically to treat patients suffered from various types of malignancies. Some of these patients are smokers, heavily exposed second-hand smokers and those using nicotine for smoking cessation or pain relief. Benzopyrene is a well-established carcinogen by forming DNA adducts and further DNA double strand breaks and co-exists with nicotine in tobacco smoking. Thus, there is an urgent need for the understanding of the influence of nicotine on DNA damage checkpoint response triggered by γ-irradiation or BP exposure. In this study, we investigated the role of nicotine on the G_1_ arrest initiated by genostress in human or murine lung epithelial cells. We showed that the activation of DNA damage response was compromised by the concurrent treatment of nicotine with γ-radiation or BP treatment, through the upregulation of cyclin D1 as well as the attenuation of Chk2 activation. However, the treatment with nicotine appeared to play no role in G_2_/M phase checkpoints. Our data strongly suggest that nicotine is capable of overriding the DNA damage checkpoint activation, thereby potentially increasing risk of cancer genesis or progression via disrupting genetic surveillance.

## Results

### Nicotine exposure interferes with DNA damage-induced G_1_/S arrest

**Figure 1:**
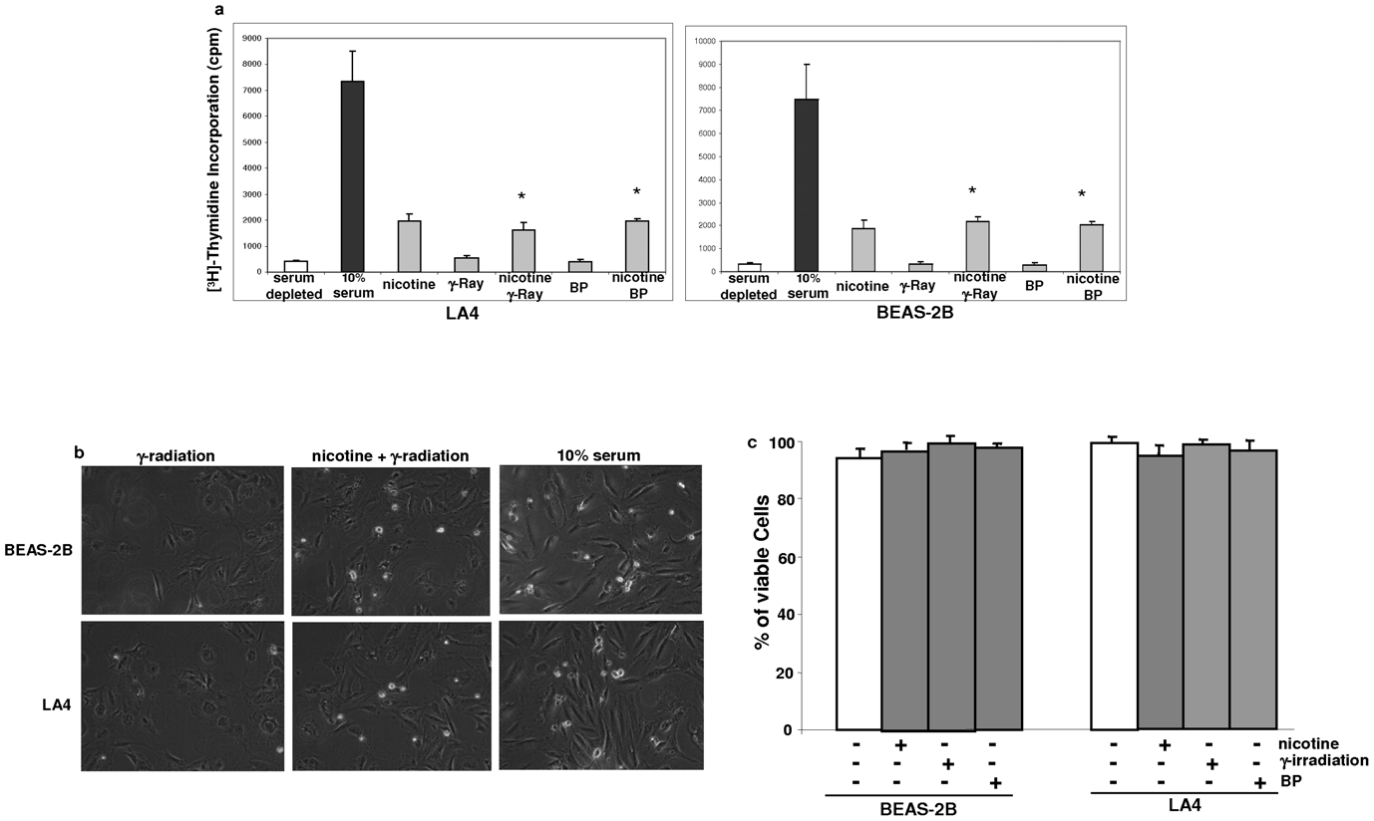
Effect of nicotine exposure on the growth of human or murine lung epithelial BEAS-2B and LA4 cells. **a**. Cells were grown in the medium containing 0.5% serum for 24 h, and then subjected to various treatments. Four hours later, the rate of [^3^H] incorporation was measured by a scintillation counter. Error bars represent the standard deviation over 5 independent experiments (n = 5, *p*<0.05). **b**. The phase contrast photos of the cells with the treatments as indicated. **c**. Four hour after the treatments as described above, Annexin V assay was performed to evaluate cell viability or occurrence of apoptosis. Error bars represent the standard deviation over 5 independent experiments (n = 5, *p*<0.05).

Nicotine is an addictive substance and has been shown to rapidly activate several extracellular, mitogenic-related pathways for growth promotion [26]–[28]. Since most of tobacco smoke components are genotoxic, we first tested the influence of nicotine on genotoxic stress in murine LA4 or human BEAS-2B lung epithelial cells. [^3^H]-thymidine incorporation assay was performed (**Fig. 1a**). After serum starvation, the cells were exposed to γ-irradiation (5 Gy), or treated with BP (benzolpyrene, 200 ng/ml) or nicotine (0.5 µM) for 4 h. Subsequently, the rate of [^3^H]-thymidine incorporation of the cells was analyzed. A very low baseline level of [^3^H]-thymidine incorporation was seen in serum starved, irradiated or BP-treated cells. The intake of [^3^H]-thymidine was dramatically upregulated after serum-starved cells were re-fed with the growth medium containing 10% serum (about 8–10 folds). In comparison, the rate of [^3^H]-thymidine incorporation was moderately increased in the cells treated with nicotine under serum starvation condition (about 3 folds). The similar effect of nicotine on [^3^H]-thymidine incorporation was observed in irradiated or BP-treated cells. The pictures of cell cultures, upon γ-irradiation in the presence or absence of nicotine, were taken by the phase contrast photography (**Fig. 1b**). Consistently, there is a few proliferating LA4 or BEAS-2B cells in the cultures. The addition of nicotine promoted some of irradiated or BP-treated cells to divide. The data suggest that nicotine partially interferes with DNA damage-induced growth arrest.

To eliminate the possibility that the lack of DNA synthesis might be due to the induction of apoptosis, the viability of the cells, after being treated with BP treatment for 4 h or exposed to γ-irradiation, was determined by Annexin V assay (**fig. 1c**). The results revealed that the majority of treated BEAS-2B or LA4 cells were viable at the time when the assay was performed, and only a few cells stained positively with Annexin V.

### Nicotine disrupts the negative effect of DNA damage on G_1_/S regulators

**Figure 2:**
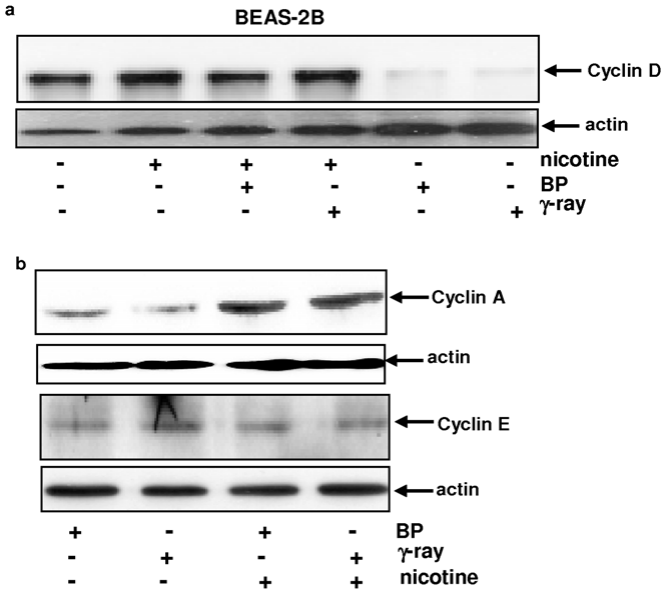
Expression of G_1_ cyclins in irradiated or BP-treated BEAS-2B cells in the presence or absence of nicotine. **a**. Cells were irradiated with γ-ray or treated with BP in the presence or absence of nicotine. Subsequently, cell lysates were prepared and immunoblotted with anti-cyclin D1 antibody. Even loading of total proteins was normalized by re-probing the nitrocellulose with anti-β-actin antibody. **b**. The expression of cyclin E or A after the same treatments as described above. Even loading of total proteins was normalized by re-probing with anti-β-actin antibody.

It is known that DNA damage causes growth arrest that is resulted from negative controls on G_1_/S regulators [24], [25]. Therefore, the effect of nicotine on DNA damage-mediated growth restriction was tested. The expression of cyclin D1, E or A was analyzed after γ-irradiation or BP treatment in the presence or absence of nicotine (**Fig. 2a**). A relatively high level of cyclin D1 was detected in BEAS-2B cells, which was slightly upregulated by nicotine treatment. The amount of the expression of this cell cycle regulator was significantly reduced after the irradiation or BP treatment (about 3.5–4 folds). However, cyclin D1 expression did not change in the cells co-treated with nicotine plus radiation or BP. The results indicate that nicotine might, via altering the expression of cyclin D1, lift DNA damage-induced growth restriction, and function as a rate limiting factor of the G_1_ restriction in nicotine-mediated cell cycle progression.

Cyclin A is an important and necessary factor in controlling the transition or progression of cells from G_1_ to S phase. Therefore, the expression of cyclin A was examined by immunoblotting (**Fig. 2b**). Again, a low level of cyclin A was present in irradiated or BP-treated cells, which was increased by the co-treatment with nicotine (about 3 folds), suggesting that cyclin A is also a rate limiting factor in nicotine-mediated interference with γ-irradiation- or BP-induced growth restriction. However, the co-treatment had no effect on cyclin E expression.

### γ-radiation- or BP-mediated Chk2 activation is blocked by nicotine exposure

**Figure 3:**
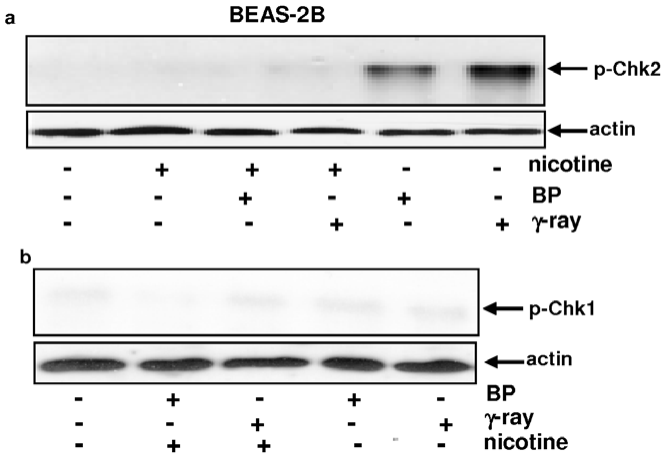
Phosphorylation status of Chk2 and Chk1 in irradiated or BP-treated BEAS-2B cells in the presence or absence of nicotine. **a**. Cells were irradiated with γ-ray or treated with BP in the presence or absence of nicotine. Subsequently, cell lysates were prepared and immunoblotted with the anti-phos-Chk2 antibody. Even loading of total proteins was normalized by re-probing with anti-β-actin antibody. **b**. The expression of the phosphorylated Chk1 after the same treatments as described above. Even loading of total proteins was normalized by re-probing with anti-β-actin antibody.

In response to genotoxic damage, Chk1 and Chk2 are phosphorylated by the upstream ATR or ATM kinase, respectively [17]–[19]. Since nicotine perturbed radiation- or BP-induced growth arrest, the phosphorylation status of Chk2 and Chk1 in BEAS-2B cells was examined by immunoblotting (**Fig. 3**). Chk2 was phosphorylated after the cells were exposed to γ-radiation or treated with BP and nicotine treatment alone played no role in Chk1 phosphorylation. However, the co-treatment of nicotine with the radiation or BP, the phosphorylated Chk2 in the cells became undetectable. The similar change of Chk2 phosphorylation after the co-treatment was also observed in murine lung epithelial LA4 cells (data not shown). In contrast, a baseline level of phosphorylated Chk1 was present in BEAS-2B cells following γ-irradiation or BP-treatment, and the co-treatment did not alter the phosphorylation pattern of this checkpoint. Thus, the data suggest that the phosphorylation of Chk2 (but not Chk1), elicited by the genotoxic stresses, was interfered by nicotine treatment.

### Nicotine treatment plays no role in nocodazole-mediated G_2_/M phase checkpoint

**Figure 4:**
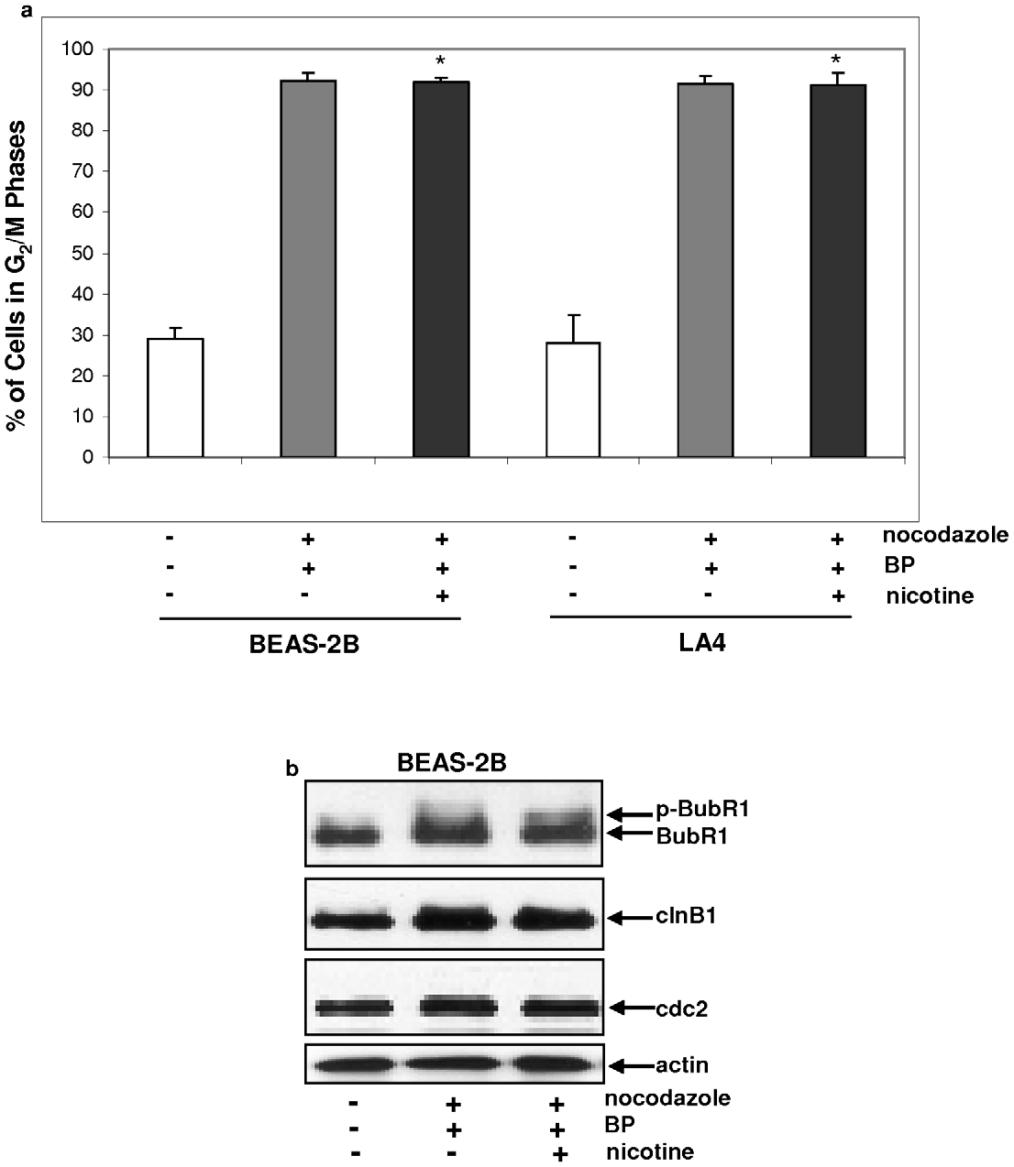
Effect of nicotine on G_2_/M phases in BP-treated BEAS-2B cells. **a**. BEAS-2B cells were treated nocodazole prior being treated with BP in the presence or absence of nicotine. Subsequently, cells were fixed and stained with propidium iodide. The percentage of G_2_/M phase population was measured by a flow cytometer. Error bars represent the standard deviation over 5 independent experiments (n = 5, *p*<0.05). **b**. After the same treatments as described above, cell lysates were prepared and immunoblotting was performed to test the expression of BubR1/p-BubR1, cyclin B1 and cdc2. Even loading of total proteins was normalized by re-probing the nitrocellulose with anti-β-actin antibody.

Nocodazole arrests cells in G_2_/M phase through disrupting microtubules of the nucleus by associating with β-tubulin and preventing the formation of the interchain disulfide linkages [29]. We tested whether the activation of nocodazole-mediated G_2_/M checkpoints upon BP treatment was compromised by nicotine. After being arrested in the M phase of the cell cycle by nocodazole, BEAS-2B or LA4 cells were treated with BP or its combination with nicotine and cell cycle distribution was measured by a flow cytometer (**Fig. 4a**). As expected, the majority of BEAS-2B cells were arrested in the G_2_/M phases of the cell cycle following the addition of nocodazole. However, the treatment with BP or its combination with nicotine had no effect on nocodazole-induced enrichment of G_2_/M population of BEAS-2B or LA4 cells.

BubR1 and cyclin B1 are key M phase regulators [30]–[32]. We next examined their activation status or expression. BubR1 was not phospohorylated in untreated BEAS-2B cells (**Fig. 4b**). After γ-irradiation, the phosphoyrlation form of BubR1 was present in nocodazole-treated BEAS-2B cells, the level of which did not change after the addition of nicotine. Consistently, upon irradiation, the level of cyclin B1 in nocodazole-treated BEAS-2B cells was higher than that in untreated cells, and nicotine treatment did not reduce the amount of cyclin B1 expression. Furthermore, the expression of cdc2 that forms a complex with cyclin B1 during G_2_/M transition was examined by immunoblotting. The similar pattern of the expression of this G_2_/M regulator as cyclin B1 was detected in irradiated BEAS-2B cells with or without nicotine treatment. It appears that nicotine had no role in M checkpoints.

### Knockdown of cyclin D1 or Chk2 abrogates the effect of nicotine on growth arrest induced by DNA damage

**Figure 5:**
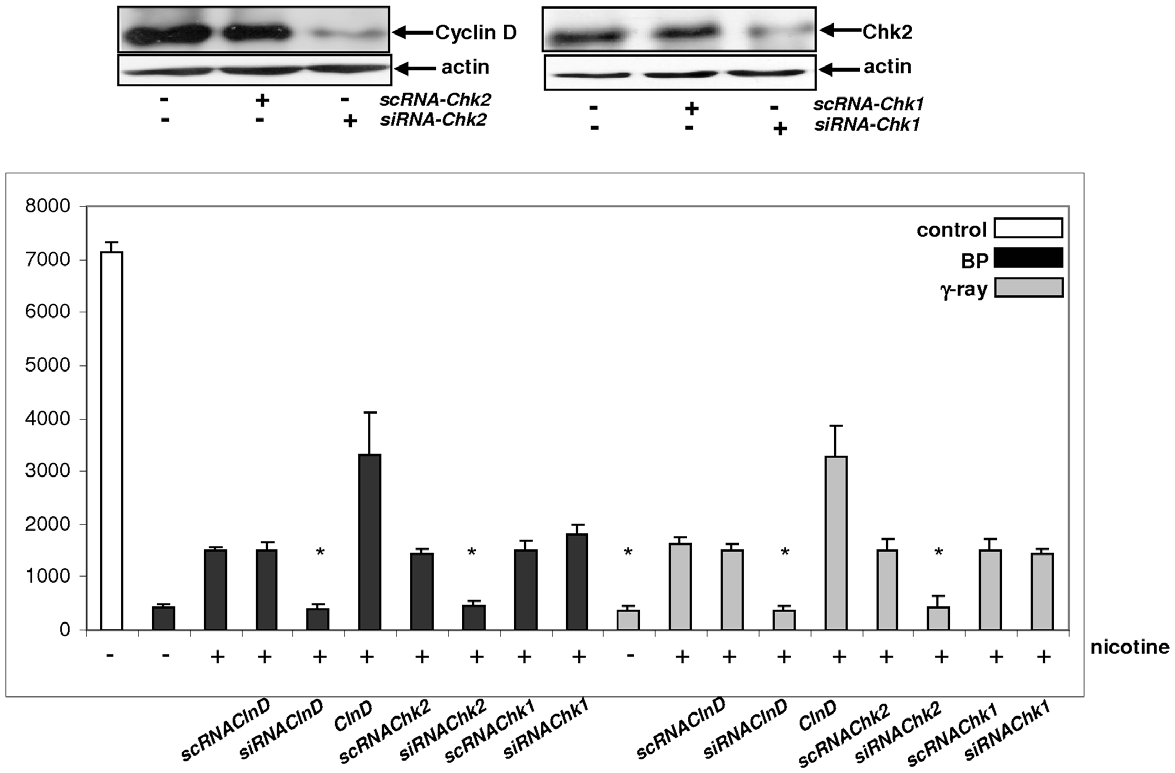
DNA synthesis of irradiated or BP-treated BEAS-2B cells after knockdown of cyclin D1, Chk2 or Chk1 upon nicotine exposure. *scRNA-, siRNA-clnD1, scRNA- or siRNAChk2* was introduced in to the cells and subsequently, the knockdown of cln D1 and Chk2 expression were analyzed by immunoblotting (upper panels). After the knockdown of *clnD1*, *Chk1* or *Chk2*, the cells with various treatments were measured for [^3^H] incorporation. Error bars represent the standard deviation over 5 independent experiments (n = 5, *p*<0.05).

The experiments demonstrated above indicated a potentially important action of nicotine in perturbing the activation of G_1_/S checkpoints induced by γ-irradiation or BP treatment. To further determine this, *shRNAs* were used and then tested the knockdown effect on DNA damage-induced growth arrest in nicotine-treated cells. *ShRNA-cyclinD1* and *shRNA-Chk2*, but not *scRNA-cyclinD1* and *scRNA-Chk2*, efficiently knocked down the expression of these two kinases in BEAS-2B cells (**Fig. 5a**). *ShRNA-Chk1* also successfully suppressed Chk1 expression (data not shown). Subsequently, the influence of nicotine on the rate of [^3^H]-thymidine incorporation in BEAS-2B cells with or without knockdown of *cyclinD1*, *Chk2* or *Chk1* or overexpression of *cyclinD1* was tested following γ-irradiation or treatment with BP (**Fig. 5b**). Again, after the exposure to γ-irradiation or BP treatment, the intake of [^3^H]-thymidine in BEAS-2B cells was almost absent. A moderate amount of [^3^H]-thymidine was incorporated into the genome of irradiated- or BP-treated cells after the addition of nicotine (3–4 folds), which was further augmented by the overexpression of *cyclinD1* (6–7 folds). In contrast, knockdown of *cyclinD1* or *Chk2* by the *shRNAs* (but not the *scRNAs*) completely blocked this nicotine-mediated [^3^H]-thymidine intake. The infection of *scRNA-Chk1* or *shRNA-Chk1* had no effect on nicotine-mediated incorporation, suggesting little role for Chk1 in the perturbation of G_1_/S checkpoints elicited by γ-irradiation or BP treatment.

## Discussion

In this study, we have investigated the mechanism by which nicotine perturbs γ-irradiation- or BP treatment-induced G_1_/S checkpoints. We showed that the addition of nicotine interfered with the inhibition of DNA synthesis induced by γ-irradiation or BP treatment, concomitant with the upregulation of cyclin D1 and A as well as abrogation of the phosphorylated form of Chk2. Furthermore, block of cyclin D1 or Chk2 signaling abolished the nicotine-mediated effect on DNA damage-induced growth restriction. These results are in agreement with our previous studies showing that long term exposure of nicotine perturbed Rb/E2F signaling pathway to promote cell proliferation [14], [33]. Using xenograft assay, other groups also demonstrated that nicotine was able to promote various lung cancer cell lines to form tumors in nude mice [12]–[14]. Collectively, our current data identified that cyclin D1 and Chk2 are intracellular targets of nicotine for the dysregulation of G_1_/S phase checkpoints activated by γ-irradiation- or BP-induced DNA damage. Further consistent with a role of the loss of the function of Chk2 in the disruption of G_1_/S checkpoints, both Chk2 phosphorylation and growth arrest induced by the radiation and BP treatment were prevented by nicotine exposure.

Nicotine is often used to aid smoking cessation or relieve chronic pain. The use of nicotine has been associated with the promotion of tumorigenesis in lung or other organs [34]. Studies demonstrated that nicotine exposure has pro-survival activity in lung epithelial cells, by activating various intracellular growth factors, such as PKC, or by upregulating Bcl-2 activity to antagonize apoptotic signals [35]. Using mouse models, studies also showed that nicotine stimulated angiogenesis in the settings of inflammation, ischemia, atherosclerosis and tumor growth [9]–[11]. Upon the interaction between nicotine and its receptor, MAP kinase activity was upregulated via a tyrosine kinase pathway. Furthermore, Akt in primary human airway epithelial cells was activated in response to transient exposure to nicotine. This effect has been suggested to contribute to tobacco-related carcinogenesis [36]. In response to persistent exposure to nicotine, cyclin D1 has been shown to be enhanced via the activation of Ras signaling and further forced cells to progress under the mitogen withdrawal condition. Our current study suggests that cyclin D1 is the primary target of nicotine that is not only involved in the promotion of cell growth but also in the disruption of G_1_/S checkpoints.

BP forms adducts with DNA that causes DNA double strand breaks through generating DNA single strand nucleotide excision repair intermediates. Thus, BP or γ-irradiation mediated DNA damage is likely to activate ATM-governed checkpoints, which was also confirmed by our findings that Chk2 was phosphorylated in response to the exposure to the radiation and BP treatment. It is possible that nicotine, through promoting cyclin D1/E2F signaling, prevents ATM/Chk2 signaling to be activated or interferes with Chk2 phosphorylation process in response to DNA double strand damage induced by either γ-irradiation or BP, and further perturbs the growth restriction.

Studies demonstrated that in nicotine-mediated growth promotion, the ligation of nicotine receptor activated Ras signaling, which further mobilized Raf/MAP kinases [32], [37], [38]. Using various promoter mutant constructs of *cyclin D1*, it was shown that AP1 site, but not *Ets* sites, was required for nicotine-induced cyclin D1 promoter activation. However, the molecular mechanisms of signaling pathways bridging nicotine-mediated cyclin D1 activation and its growth promotion are not fully understood yet. Here, we demonstrated the deregulation of cyclin D1, A and Chk2 by nicotine in irradiated or BP-treated lung cells. It is conceivable that different mechanisms for the activation of cyclins and suppression of Chk2 activity are involved in nicotine-mediated growth promotion and perturbation of G_1_/S checkpoint induced by DNA damage (such as γ-irradiation and BP treatment). The studies of the underlying mechanisms are under way.

Our current study provided a particularly interesting observation that the exposure to nicotine appears to compromise the activation of G_1_/S checkpoints elicited by γ-irradiation or BP treatment, but not to G_2_/M checkpoints. Clinical and epidemiological studies suggest that first-hand or heavily exposed second-hand smokers have more progressive and metastatic cancers than that in non-smokers [2]. The incidences of the metastasis of breast cancers to the lung in smokers are much higher than non-smokers [11]. Our current data suggest that the alteration of G_1_/S checkpoint response to DNA damage by nicotine is highly relevant in this context. Indeed, others have demonstrated that persistent exposure to nicotine resulted in the downregulation of E-cadherin and β-catenin with concomitant increase of fibronectin and vimentin [11]. It has also been reported through clinical studies that smoking reduced the expression level of E-cadherin in lung tumors, which might be responsible for the establishment of the resistance to conventional chemotherapy [11]. From our study, it is conceivable that apart from the role in epithelial-mesenchymal transition or metastasis, this cigarette component takes part in the perturbation of DNA damage-regulated checkpoint response triggered by BP (one of carcinogens in tobacco smoking) or by γ-radiation.

In summary, we have identified a new mechanism by which nicotine interferes with DNA damage-induced G_1_/S checkpoints. Our study also provided an insight into the cooperation of the components of cigarette smoking in lung carcinogenesis, because a high concentration of nicotine co-exists with various tobacco carcinogens, such as BP. Our findings also warrant the mechanism for cancer patients who are smokers, heavily exposed second-hand smokers or nicotine users to develop the resistance to radiation therapy.

## Materials and Methods

### Cell lines and treatments

All cell lines were obtained from ATCC (the American Type Culture Collection, Mamassas, VA). The cells were cultured in the medium according to the protocols provided by ATCC. Benzopyrene and nocodazole were purchased from Sigma.

### DNA synthesis analysis

Cells were cultured in 60-mm Petri dishes in the presence of 15 nCi/ml [^14^C] thymidine (Amersham) to label DNA for 48 h. The cells were fed with fresh medium containing [^14^C] thymidine for another 24 h. Subsequently, the cells were irradiated or treated with DNA damage agents. One hours after irradiation or treatment, the cells were cultured in fresh medium containing [^3^H]-methyl thymidine (5 µCi/ml) (New England Nuclear) for 4 h. Afterward, the cells were washed with ice-cold 5% trichloro-acetic acid and solubilized in 0.3 N NaOH. The solubilized material was transferred to a scintillation vial and neutralized with 0.1 ml of glacial acetic acid. The ^3^H and ^14^C radioactivity in the samples was measured by dual-channel liquid scintillation counting (49). The relative rates of DNA synthesis were determined by ^3^H cpm: ^14^C cpm ratio.

### Colony formation assay

After radiations or treatments, cells (500 cells/per dish) were plated in the 100 mm-Petri dishes and cultured in the growth medium for 10–14 days. Colonies were stained with Giemsa (Sigma).

### Cell Cycle Analysis

Cell cycle distribution of DNA content was measured using a flow cytometer as described (50). The cells, after the treatments, were fixed in 65% DME medium and 35% ethanol for 2–4 h at 4°C and stained with propidium iodide. Subsequently, the DNA profiles were analyzed using Cell Quest software.

### Immunoblotting analysis

After treatments, proteins in cell lysates were separated by SDS-PAGE gels and then transferred to nitrocelluloses. The membranes were incubated with the designated primary antibody overnight in a cold-room at 4°C. Bound primary antibodies were reacted with corresponding second antibodies for 2 h and detected by chemiluminescence.

### Annexin V-FITC apoptosis detection assay

After treatments, cells were prepared and stained with Annexin V-FITC Apoptosis Detection Kit I (BD Biosciences) according to manufacturer's instructions. Subsequently, the samples were analyzed by a flow cytometer.

### Reproducibility

All data are representative of experiments that were performed for more than three times.

### Statistics

Three to five independent repeats were conducted in all experiments. Error bars represent these repeats. A Student's T test was used and a *p* value of <0.05 was considered significant.
